# Late Effect of the Cervical Irradiation on Periodontal Status and Cariogen Flora in Hodgkin Lymphoma Patients

**DOI:** 10.5402/2011/823926

**Published:** 2011-03-06

**Authors:** Zsófia Simon, Ildikó Tar, Katalin Gáll, Borbála Ivancsó, Judit Szabó, Árpád Illés

**Affiliations:** ^1^3rd Department of Institute for Internal Medicine, University of Debrecen, 4032 Debrecen, Hungary; ^2^Department of Periodontology, Faculty of Dentistry, University of Debrecen, 4032 Debrecen, Hungary; ^3^Faculty of Dentistry, University of Debrecen, 4032 Debrecen, Hungary; ^4^Institute of Microbiology, Medical and Health Science Center, University of Debrecen, 4032 Debrecen, Hungary

## Abstract

Cervical radiotherapy may leads to elevated caries risk in Hodgkin-lymphoma (HL) patients. Our aim was to estimate the late effect of cervical irradiation on periodontal status in HL patients. Patients filled out query-form, their clinical data were collected, periodontal status was examined, decayed-missing-filled-teeth and periodontal-indexes were calculated. We examined 68 patients who received, 64 patients who did not received cervical radiotherapy and 51 control person. 23.5% of cervical irradiated, 18.15% of not irradiated patients and 17.64% of controls had subjective xerostomia, but it was not objective by sialometry. Mean decayed-missing-filled-teeth-index was 22.53 among irradiated, 21.54 among not irradiated patients while it was 17.23 in control group. Periodontal index was 2.47, 2.42, and 2.14 in different groups. Difference between decayed-missing-filled-teeth indexes of irradiated patients and controls was significant. We have to emphasize the importance of prevention and closer dental observation of HL patients.

## 1. Introduction

In the last decades the results of the treatment of Hodgkin lymphoma (HL) patients have improved, they are cured, and now we have to be faced with the late complications of the lymphoma and/or the treatment of these, which can both influence the long-term survival and the quality of life of the patients. The radiotherapy has played an important role in the treatment of HL since the first successes till these days, but now it is well known that beside its favorable effects the radiotherapy is liable for most of the serious therapeutic side effects and the late complications (secunder malignancies, cardiovascular diseases, irradiation pneumonitis and fibrosis, hypothyreosis, etc.) [1–3]. The extended field (mantle-field and total-nodal) irradiation frequently used earlier and the field irradiation involving the upper cervical region most commonly used nowadays can damage the salivary glands beside the active antitumor effect [4–6]. The influence of the radiotherapy on the saliva flow was investigated in patients with head and neck tumor. This side effect is less known in lymphoma patients, and it is less investigated what kind of late complications the irradiation has on the salivary gland function and on the periodontal status of them.

In the treatment of head and neck tumors the irradiation often gets important role, and it may lead to the damage of speaking and chewing functions and/or to the decrease of the salivary outcome, the development of xerostomia caused by the damage of salivary glands [4, 5]. After the irradiation mostly the serous glands and acini are injured, fibrosis may develop in the patients, the salivary production decreases, and the saliva becomes sticky and viscid. If the irradiation dose is below a critical limit, the patients' saliva flow can recover after 18–24 months [4, 7–10]. Beside its role in chewing and digestion, the saliva gives a kind of protection against microorganisms. This is a physical and chemical protection that is not specific except its specific immune components. There is a thin saliva coat on the oral serosa with limasol property what leads to water adsorption. The matter of the physical protection is that the adsorbed water is not wetting the epithelium so the permeability against bacteria decreases. The antibacterial products of the saliva (lisosim, peroxidase-thiocyanate system, lactoferrin, IgA) are liable for the chemical protection. It can be seen that the saliva pays an important role in the reservation of the healthy milieu of the oral cavity. Its decreased production may lead to an increase of the cariogenic oral flora, and this may result in a deteriorated dental and periodontal status. Beside the decreased saliva flow caused by the irradiation in the case of HL patients, the associated cellular immunodeficiency and the strong immuno- and myelosuppression caused by the chemotherapy also promote the proliferation of the cariogenic oral flora. All this may result in activation of local inflammations, periodontitis, or soor mycosis, and moreover the irradiation may lead to the destruction of the dental alveoli, osteoradionecrosis of (mostly the) mandibula, and destroy of the dental germs, which implicitly can be a serious complication especially in the childhood cases [11].

On the basis of the above we decided to investigate in our HL patients whether the radiotherapy involving the salivary glands has a long-term influence on the saliva production, and the side effects of the antitumor therapy reflect in the periodontal status of our patients.

## 2. Patients and Methods

We investigated 132 HL patients attended in the 3rd Department of Internal Medicine, University of Debrecen, and 51 control persons matched in gender and age, who had no tumor or chemo-/radiotherapy in his/her medical history. The HL patients during their treatment received only radiotherapy (RT), only chemotherapy (CT), or the combination of them (CMT), and we divided them into two groups based on whether the upper cervical region was irradiated or not. The control persons formed the third group. During the selection of the investigated persons (either the patients and the members of the control group), we excluded those who had habits or diseases or medication what can influence the saliva production (primer immunodeficiency, AIDS, I or II type diabetes mellitus, Sjögren's syndrome, gravidity, hypothyreosis needs substitution, *β*-blockers, atropine, diuretics, hydantion treatment, smoking). To survey the case histories and the detailed treatment of the patients, we used the MedSolutions medical software data base and the medical charts.

HL patients mostly received COPP (cyclophosphamide, vincristin, procarbazin, prednisolone), COPP/ABV (cyclophosphamide, vincristin, procarbazin, prednisolone, adriablastin, bleomycin, vinblastin), or recently ABVD (adriablastin, bleomycin, vinblastin, dacarbazin) chemotherapy. Formerly we used extended field (mantle, inverse Y, total nodal) irradiation in 40–45 Gy cumulative dose, while in the last decade there was only 30–36 Gy cumulative dose in the involved field.

We examined the patients in the Department of Periodontology, where first we recorded the history data (former diseases, dental interventions, medication, smoking habit, subjective xerostomia) by a *questionnaire*, then we did sialometry and examined simple general dental and periodontal status, measured the buffer capacity of the saliva, and made microbiological sampling to the examination of the cariogenic flora. One hour before the examination, the patients were not allowed to eat and drink. 

By *sialometry* we measured the five minutes unstimulated, then after five minutes the stimulated saliva production in a sialometric tube (after a five-minute long examination the saliva production is considered as stimulated). We considered the amount of less than 0.1 mL/min saliva as pathological.

We measured the *buffer capacity of the saliva* by CRT semiquantitative strip.

To feature the *dental status* we used the DMFT index what shows the rate of *d*ecayed, *m*issing, and *f*illed teeth.

To survey the *periodontal status *we used the simplified Russell periodontal index by examining the status of the gum and the periodontium on four surfaces of every teeth (mesial, distal, facial, lingual). By physical examination we measured the blow-pipe depth and presence of gingivitis or plaque. The median rate of the measured values gave the index of one tooth, and the arithmetical average of these gave the person's periodontal index.

To examine the *cariogenic flora* we got some samples from the lingual surface of the lower incisors with a special sterile stick, and we carried it in transport medium to the Institute of Microbiology, where the samples were plated. *Streptococcus mutans*, *Lactobacillus* sps., and *Candida albicans* were bred, and in the case of positive breeding the number of the colony forming units (CFU) also were counted. The presence of the causative agent was considered as significant if it achieved the order of 10^5^ after 48–72 hours of breeding. The *S. mutans* was bred on selective mitis salivarius agar substrate (Becton Dickinson) on 37°C in 5% CO_2_ milieu, while the *Lactobacillus* sps. was bred on Rogosa agar substrate (Merck) on 37°C between anaerob conditions. The *Candida albicans* was bred on Sabouraud glucose-agar substrate (Oxoid) and on chromagar (Becton Dickinson). The identification of *S. mutans* at species level was done by RapidID32 Strep (bioMerieux, Durham, N.C.), the Lactobacilli at genus level by API 50 CH (bioMerieux, Durham, N.C.), and the *Candida albicans* at species level by ID32C (bioMerieux, Durham, N.C.) automatic identifier panel.

The statistical analysis was made by StatSoft (Version 6.) software with Lilliefors and Kolmogorov-Smirnov tests. In the case of parametric distribution, we used T-probe, and in nonparametric distribution we applied Mann-Whitney test. If we compared more than two series, the *P* value was calculated with ANOVA test, and we considered it as significant if *P* value was <.05.

## 3. Results

Among the 132 HL patients, 68 (32 female and 36 male) received irradiation including the upper cervical region, and beside the radiotherapy 54 patients got chemotherapy as well. Their average age was 47.1 (20–79) years, the mean follow-up time was 12.8 (2–37) years after the treatment. 64 HL patients received only chemotherapy, or beside the chemotherapy they got irradiation not including the cervical lymph nodes. In this group the average age of patients was 44.05 (21–75) years, and 11.7 (2–34) years had passed since the treatment. The average age of the 51 control persons (25 female, 26 male) was 45.8 (20–68) years. The data of these groups are shown in Table 1. 

In all three groups the 1/5 of the patients had subjective xerostomia, but we were not able to make it objective by sialometry as the saliva flow was at least 0.1 mL/min in every case, which is the normal level. In both HL groups the average value of the salivary outcome was less than it was in the control group, but the difference was not significant. The buffer capacity of the saliva was the lowest in the cervically irradiated group, but the alteration was not significant either. The DMFT index and the periodontal index were the worst in the cervically irradiated HL patients group and the difference between the DMFT of the irradiated HL patients and the control group was significant (Table 2). Examining the cariogenic flora in the sample collected from the dental surface, all the three pathogens were detectable in the same ratio in the different groups of patients. However, the mostly cariogenic *S. mutans* was present in the highest ratio (73.52%) with a significant germ amount in the sample of cervically irradiated patients; it was 65.5% in the not irradiated group and 60.8% in the control group. The differences were not significant.

## 4. Discussion

In the past decades the changes in the treatment of Hodgkin lymphoma led to long-term survival of the patients; 70%–80% of them are cured. Parallelly with this, we had to face the late side effects of the treatment, among which the second malignancies and the higher cardiovascular risk are the most serious. The first smashing success in the HL therapy was the application of irradiation, and, although nowadays we rarely use the irradiation alone beside the effective chemotherapy, it is still indispensable. The side effects of radiotherapy are also well known (e.g., dermatitis, mucositis, esophagitis, pneumonitis, etc.). In the present study we analyze the late complication of the salivary glands' irradiation damage, which has been examined in this group of patients less often. In the treatment of HL the extended field irradiation had been used the most commonly before the last ten–fifteen years, and among these the total nodal and the mantle field irradiation both involved the cervical lymph nodes and the submandibular and sublingual salivary glands and a part of the parotis with them. Nowadays, by using modern radiotherapy with the protection of the surrounding healthy tissues, only the involved lymph nodes are irradiated with a reduced dose, so the salivary glands are rarely damaged (e.g., upper cervical lymph nodal region) [1–4].

The cariea formation is a multicausal course and in its development the dietetic habits, the microorganisms of the plaque, the dental surface, and other etiological factors either can play role. The proliferation of the cariogenic organs, the unfavorable quantitative and qualitative changes of the saliva are among the risk factors of caries formation.

Beside the hypothyreosis, the xerostomia is one of the most common complication of the upper cervical irradiation. It is caused by the development of fibrosis and atrophia and the damage of the serosal salivary glands and acini, which are more sensitive to the ionic radiation. The amount of the saliva decreases, its viscosity increases, its electrolyte content is modified, and both the quantitative and the qualitative changes are favorable to the proliferation of the cariogenic flora.

Franzen et al. [8] and Someya et al. [4] found that decrease of the saliva flow correlates with the cumulative radiation dose in head and neck tumor patients. Under the limit of maximum 50–52 Gy cumulative, dose the decrease of saliva outcome can be reversible being better two months after the treatment and recovering completely in an 18–24 month period, but, using more than 58–64 Gy cumulative dose, the damage is surely irreversible. In the course of Hodgkin lymphoma's treatment, the cumulative irradiation dose comes at the critical 50 Gy level only in a few cases, so the chronic damage of the saliva glands is very rare. We have seen it in our work too: although 1/5 of the patients had subjective xerostomia, we were not able to make this objective in any of them. The increased risk of caries formation during and after the irradiation is well known for a long time. The oral flora will be cariogenic because of the proliferation of the *S. mutans*, *Lactobacillus* sps., and the *C. albicans*. As soon as at the beginning of the treatment, Keene at al. observed the unfavorable proliferation of the microorgans parallelly with which the dental status was decreasing in patients with Hodgkin lymphoma or head and neck tumor. [5, 6]. At the MD Anderson Cancer Center in Houston, the daily self-applied topical fluoride gel has been recommended since 1966 for head and neck cancer patients before, during, and after radiotherapy. The study of Keene et al. confirmed that, by using this prevention protocol, the increase of the most cariogenic *S. mutans* and the caries formation would be significantly less, even though just with a small number of patients [5, 6]. In this study the oral flora was analyzed during the radiotherapy and soon after that. In our paper we observed that, although neither the cariogenic oral flora nor the periodontal index show significant differences between the altering groups, the general dental status was significantly worse in the cervically irradiated patients than in the control group. This allowed us to conclude that, even though the decrease of the salivary flow and the proliferation of the oral flora are reversible processes, the damages developing during the treatment significantly worsen the HL patients dental status in a long term, and the irradiation plays an important role in this.

Among the HL patients there is a higher predisposition to the proliferation of pathogen micro-organs, development of opportunistic infections, what can be caused by the strong immunosuppressive therapy, and the consequential neutropenia beside the decreased cellular immunity characteristic to the HL. However, it is worth mentioning that some kinds of cytostatic drugs (methotrexate, doxorubicin) diminish the number of *S. mutans* and Lactobacilli during the antitumor treatment as a Finnish working group observed [12], but a long-term follow-up study is missing. With the chemotherapy against the HL (COPP, COPP/ABV, BEACOPP), the patients get high dose of steroid, what has an immunosuppressive effect, but beside that it is able to provoke the development of diabetes mellitus, which is also subservient for the proliferation of pathogens. We closed out the patients with diabetes from this study, but this group needs special attention as for the prevention.

The irradiation beside the higher caries risk may lead to the destruction of the dental alveoli, osteoradionecrosis, which mostly develops in the mandibula with a worse blood flow. This complication can be prevented with the extraction of the incurable tooth before the treatment, which emphasizes the importance of the pretreatment screening by the dentist [11]. The radiotherapy makes the patient liable to develop second malignancies, and of course by these the oral cavity also can be affected, so the regular dental checking visits can help a lot to screen them out.

As a summary we can say that the irradiation has an unequivocally unfavorable effect on the salivary flow and the oral flora, which means a higher cariogenic risk. This can be an infection gate during the therapy of a patient with malignancy; for a shorter or longer time, the xerostomia, the wrong dental and periodontal status for a lifetime can worsen the quality of life. That is why it is relevant to emphasize the importance of the increased oral hygiene and the regular visits at the dentist. 

Beside using a special fluoride gel and a conventional toothbrush, rinsing with chlorhexidine, the routinely use of other special aids can be recommended, for example, dental floss, special interdental toothbrush, and oral shower. The xerostomia can be favorably influenced by saliva flow stimulating products, even by using prosthetic saliva, or as alternative medication vegetable bitter matters and occurrently acupuncture can be applied. As soon as during the first treatment we have to start the effective prevention of the long-term dental side effects.

## Figures and Tables

**Table 1:** General characteristics of the patients and control persons. Comparative data of the treatment of 132 Hodgkin lymphoma patients. RT: radiotherapy, CMT: combined modality treatment.

Characteristics	Groups				
	Hodgkin lymphoma				Control
	Cervical irradiated 68 patients		No cervical irradiation 64 patients		51 persons
Age (years)	47.1 (20–79)		44.05 (21–75)		45.8 (20–68)
Gander (male/female)	36/32		31/33		26/25
Follow-up time from the treatment (years)	12.8 (2–37)		11.7 (2–34)		—

Treatment mode					

Only RT	14	38 Gy (30–45)	2	35 Gy (20–40)	—
CMT	54		25		—
Only chemotherapy	—		37		—

**Table 2:** Results of the examination of the saliva flow and the dental, periodontal status among our cervical irradiated and not irradiated Hodgkin lymphoma patients and the control persons. (RT: radiotherapy, DMFT: decay, missing, filled tooth, NS: not significant *cervical irradiated versus control).

Methods	Group			
	Hodgkin lymphoma		Control group	Significance
	Cervical irradiated	No cervical irradiation		
Subjective xerostomia (%)	23.5	18.15	17.64	NS
Sialometry (average mL/5 min)	2.7 (1–7)	2.75 (1–8.5)	3.4 (1.5–8)	NS
Decreased saliva buffer capacity (%)	32.35	28.12	25.49	NS
DMFT index	**22.53 **(13–32)	21.54 (12–31)	**17.23 **(5-28)	*P* < .05*
Periodontal index	2.47 (1.52–4)	2.42 (1.34–3.67)	2.14 (0.37–4)	NS

## References

[1] Henry-Amar M, Somers R (1990). Survival outcome after Hodgkin’s disease: a report from the international data base on Hodgkin’s disease. *Seminars in Oncology*.

[2] Miltenyi Zs, Keresztes K, Vegh J (2007). What is the price of survival in Hodgkin’s lymphoma?. *Hematological Oncology*.

[3] Küppers R, Yahalom J, Josting A (2006). Advances in biology, diagnostics, and treatment of Hodgkin’s disease. *Biology of Blood and Marrow Transplantation*.

[4] Someya M, Sakata KI, Nagakura H, Nakata K, Oouchi A, Hareyama M (2003). The changes in irradiated salivary gland function of patients with head and neck tumors treated with radiotherapy. *Japanese Journal of Clinical Oncology*.

[5] Keene HJ, Fleming TJ (1987). Prevalence of caries-associated microflora after radiotherapy in patients with cancer of the head and neck. *Oral Surgery, Oral Medicine, Oral Pathology*.

[6] Keene HJ, Fleming TJ, Toth BB (1994). Cariogenic microflora in patients with Hodgkin’s disease before and after mantle field radiotherapy. *Oral Surgery, Oral Medicine, Oral Pathology*.

[7] Terman P, Lacher MJ (1990). Dental complications after treatment for Hodgkin’s disease. *Hodgkin’s Disease*.

[8] Franzen L, Funegard U, Ericson T, Henriksson R (1992). Parotid gland function during and following radiotherapy of malignancies in the head and neck. A consecutive study of salivary flow and patient discomfort. *European Journal of Cancer*.

[9] Stone HB, Coleman CN, Anscher MS, McBride WH (2003). Effects of radiation on normal tissue: consequences and mechanisms. *Lancet Oncology*.

[10] Cooper JS (1995). Late effects of radiation therapy in the head and neck region. *International Journal of Radiation Oncology Biology Physics*.

[11] Bras J, De Jonge HKT, Van Merkesteyn JPR (1990). Osteoradionecrosis of the mandible: pathogenesis. *American Journal of Otolaryngology*.

[12] Meurman JH, Laine P, Murtomaa H (1991). Effect of antiseptic mouthwashes on some clinical and microbiological findings in the mouths of lymphoma patients receiving cytostatic drugs. *Journal of Clinical Periodontology*.

